# Essential Role of Sperm-Specific PLC-Zeta in Egg Activation and Male Factor Infertility: An Update

**DOI:** 10.3389/fcell.2020.00028

**Published:** 2020-01-29

**Authors:** Alaaeldin Saleh, Junaid Kashir, Angelos Thanassoulas, Bared Safieh-Garabedian, F. Anthony Lai, Michail Nomikos

**Affiliations:** ^1^Member of QU Health, College of Medicine, Qatar University, Doha, Qatar; ^2^College of Medicine, Alfaisal University, Riyadh, Saudi Arabia; ^3^Department of Comparative Medicine, King Faisal Specialist Hospital and Research Center, Riyadh, Saudi Arabia; ^4^School of Biosciences, Cardiff University, Cardiff, United Kingdom; ^5^Biomedical Research Center, Qatar University, Doha, Qatar

**Keywords:** sperm, phospholipase C zeta, PLC zeta, egg activation, fertilization

## Abstract

Sperm-specific phospholipase C zeta (PLCζ) is widely considered to be the physiological stimulus responsible for generating calcium (Ca^2+^) oscillations that induce egg activation and early embryonic development during mammalian fertilization. In the mammalian testis, PLCζ expression is detected at spermiogenesis following elongated spermatid differentiation. Sperm-delivered PLCζ induces Ca^2+^ release via the inositol 1,4,5-trisphosphate (InsP_3_) signaling pathway. PLCζ is the smallest known mammalian PLC isoform identified to date, with the simplest domain organization. However, the distinctive biochemical properties of PLCζ compared with other PLC isoforms contribute to its unique potency in stimulating cytosolic Ca^2+^ oscillations within mammalian eggs. Moreover, studies describing PLCζ “knockout” mouse phenotypes confirm the supreme importance of PLCζ at egg activation and monospermic fertilization in mice. Importantly, a number of clinical reports have highlighted the crucial importance of PLCζ in human fertilization by associating PLCζ deficiencies with certain forms of male factor infertility. Herein, we give an update on recent advances that have refined our understanding of how sperm PLCζ triggers Ca^2 +^ oscillations and egg activation in mammals, while also discussing the nature of a potential “alternative” sperm factor. We summarise PLCζ localization in mammalian sperm, and the direct links observed between defective PLCζ protein in sperm and documented cases of male infertility. Finally, we postulate how this sperm protein can be used as a potential diagnostic marker, and also as a powerful therapeutic agent for treatment of certain types of male infertility due to egg activation failure or even in more general cases of male subfertility.

## Sperm PLCζ is the Primary Stimulus for Egg Activation and Early Embryonic Development

In mammalian fertilization, the fertilizing spermatozoon stimulates egg activation, a fundamental event that initiates embryonic development (Nomikos et al., 2017a). It is well established that the most crucial event of egg activation is an acute increase in cytosolic free Ca^2+^ concentrations, which in mammals occurs in the form of long-lasting Ca^2+^ oscillations that commence at or directly following gamete fusion, and persist for several hours beyond meiotic completion (Stricker, 1999; Malcuit et al., 2006; Kashir et al., 2013a). This Ca^2+^ signaling paradigm is essential for the completion of the multiple events of egg activation. However, previous archetypes of our understanding regarding the distinct events of egg activation and the events controlling them are continuously being unraveled and questioned, with specifics still being investigated. It is, however, clear that Ca^2+^-release is an integral component of egg activation in all species studied to date (Cran et al., 1988; Swann and Ozil, 1994; Jones, 1998; Nomikos et al., 2012; Limatola et al., 2019b). Over the last few decades, a number of sperm-derived molecules had been proposed as potential soluble sperm factors responsible for the generation of Ca^2+^ oscillations during mammalian fertilization (for more information see Nomikos et al., 2012, 2013a, 2017a). The fact that sperm-induced Ca^2+^ oscillations are caused by activation of the inositol 1,4,5-trisphosphate (InsP_3_) signaling pathway (Miyazaki et al., 1992) suggested that the sperm factor might itself be a phospholipase C (PLC) isoform (Jones et al., 1998).

In 2002, a novel testis-specific PLC, termed PLC zeta (PLCζ), was discovered (Saunders et al., 2002) and abundant experimental evidence has accumulated over the years suggesting that PLCζ fulfills all prerequisite criteria of the soluble sperm factor responsible for the generation of Ca^2+^ oscillations at mammalian fertilization (Cox et al., 2002; Saunders et al., 2002, 2007; Knott et al., 2005; Kouchi et al., 2005; Nomikos et al., 2005, 2013b, 2017a; Swann et al., 2006; Yu et al., 2008; Kashir et al., 2012a). Upon sperm-egg fusion, PLCζ is proposed to be delivered by the fertilizing sperm into the ooplasm, triggering the Ca^2+^ oscillations via the InsP_3_ signaling pathway, through the hydrolysis of its membrane-bound phospholipid substrate, PIP_2_ (Saunders et al., 2002; Nomikos, 2015). The importance of this sperm specific protein in mammalian fertilization has been further highlighted by numerous clinical studies directly linking defects or deficiencies in human PLCζ with documented cases of male factor infertility (Yoon et al., 2008; Heytens et al., 2009; Nomikos et al., 2011a, 2017b; Kashir et al., 2012b, c; Escoffier et al., 2016; Torra-Massana et al., 2019).

Intriguingly, two recent independent studies described the phenotype of a PLCζ “knockout” mouse (Hachem et al., 2017; Nozawa et al., 2018). By using multiple transgenic models of PLCζ “knockout” mice generated by CRISPR/Cas methodology, both studies reported that males can produce offspring, albeit with significantly reduced litter numbers (∼25%). Interestingly, both studies showed that sperm lacking functional PLCζ protein failed to induce Ca^2+^ release when microinjected into mouse eggs by ICSI. However, *in vitro* fertilisation (IVF) with such sperm, produced atypical and delayed patterns of Ca^2+^ oscillations (lower in number and frequency) with a high degree of polyspermy and activation failure, compared to the robust, physiological pattern triggered by physiological PLCζ-induced egg activation (Nozawa et al., 2018; Satouh and Ikawa, 2018).

Perhaps the atypical and delayed pattern of Ca^2+^ release, observed alongside the low number of embryos and offspring, could be spontaneous activation, unrelated to Ca^2+^ release, which is common in some strains of mice (Cheng et al., 2012), alongside with the introduction of PLCζ knockout sperm. Indeed, eggs that had been fertilized by knockout sperm also displayed multiple pronuclei, consistent with the inability of a sufficient polyspermy block (Nozawa et al., 2018). Critically, however, eggs fertilized with PLCζ knockout sperm exhibited a total of 3–4 oscillations in total, initiating following a 1-h delay. This was in contrast to normal fertilization where 3–4 oscillations were observed per hour over 3–4 h (Nozawa et al., 2018; Satouh and Ikawa, 2018). Such observations perhaps suggest that sperm containing a second molecule with Ca^2+^ releasing activity, albeit weaker than PLCζ (Jones, 2018). From such results, one could potentially posit that perhaps PLCζ is not an absolute requirement for natural fertilization, and that perhaps an alternative “primitive” or “cryptic” sperm factor may also be involved in leading to egg activation (Nozawa et al., 2018; Satouh and Ikawa, 2018).

It is possible that such a factor could be one of the previously proposed unsuccessful candidates for the “sperm factor,” including tr-kit (Sette et al., 2002), citrate synthase (Harada et al., 2007), or PAWP (Aarabi et al., 2010), which while not contributing to the majority of Ca^2+^ release at oocyte activation, may have a contributory function, especially, in the absence of PLCζ. However, it is worth noting that none of the aforementioned proteins is able to elicit Ca^2+^ release in the specific manner required for oocyte activation at physiological levels within sperm (Kashir et al., 2014; Nomikos et al., 2014; Satouh et al., 2015), while none of the alternatively proposed sperm factors (apart from PLCζ) has been shown to be directly involved in IP_3_-mediated Ca^2+^ release (Kashir et al., 2014). Furthermore, we cannot exclude the possibility that another sperm-associated enzyme, which might be only able to achieve critical levels due to absence of PLCζ in the sperm of PLCζ knockout mice, might play the role of the “cryptic” factor triggering embryogenesis by a distinct mechanism.

Theories regarding RNA involvement are also questionable since the total amount of PLCζ RNA present within sperm may not be enough to elicit any Ca^2+^ release. On the other hand, this may have been altered as part of genetic compensation.

Intriguingly, starfish eggs pre-injected with heparin (which also blocks InsP_3_ receptor function) to disrupt cytoskeletal arrangement were unable to exhibit a rapid Ca^2+^ wave response upon interaction with sperm, instead exhibiting a much more delayed pattern of release, and failed to prevent polyspermy. Furthermore, the amplitude of subsequent Ca^2+^ peaks were reduced, exhibiting an effect similar to observations made with sperm from PLCζ-null mice. In starfish, it was suggested that heparin- or age-induced hyperpolymerization of the cortical actin disrupted actin cytoskeleton dynamics at fertilization influenced Ca^2+^ release (Puppo et al., 2008; Santella et al., 2015; Limatola et al., 2019a), potentially also impacting upon subsequent events in egg activation such as cortical granule exocytosis. It is thus possible that due to the lack of a sufficient response at fertilization due to deficient/absent PLCζ, a similar effect was observed in the PLCζ-null mice, where similar symptoms of insufficient Ca^2+^ release and increased polyspermy were also observed. Indeed, it may be the case that the low number and frequency of Ca^2+^ observed could be due to events surrounding actin polymerization or associated InsP_3_-independent events of Ca^2+^ release (such as influx). However, these mechanisms are poorly understood in mammals, and require further investigation to fully ascertain.

It is clear that both Hachem et al. (2017) and Nozawa et al. (2018) represent keystone studies that support the notion that PLCζ is the primary physiological stimulus that triggers the required specific pattern of Ca^2+^ oscillations, ensuring monospermy and eventually successful egg activation and early embryonic development (Hachem et al., 2017; Nozawa et al., 2018; Swann, 2018). Moreover, the presence of an alternative factor in other species and especially in humans is still questionable, particularly taking into consideration all the documented cases of male factor infertility due to PLCζ deficiencies. However, this is an intriguing area of investigation that ongoing studies are now aiming to address. It would be interesting to examine how the increasing body of invertebrate animal work will direct the mammalian side of the coin in the future, particularly with relation to the early influence exerted by the egg actin cytoskeleton upon patterns of Ca^2+^ release and fertilization as is being unraveled in starfish. Furthermore, integral studies are required in particularly livestock mammalian models to demonstrate whether PLCζ-loss resembles the mouse and/or human scenarios. Perhaps of particular interest should be attempts to generate transgenic knockout models of PLCζ in porcine or bovine systems. While this would of course be considerably harder to perform than in the mouse, such data would undoubtedly assist in ascertaining the validity of “cryptic factor” theories. Further experiments that would be prudent would be to examine the specific timing of the reduced profiles of Ca^2+^ release in relation to PLCζ knockout sperm-egg fusion and fertilization. Are such reduced frequency and amplitude oscillations due to fusion of a single sperm, or the cumulative effect of multiple sperm-egg fusion events? It is necessary that such experiments are performed to ascertain fully the conflicting data generated from knockout studies thus far.

## Sperm PLCζ Structure and Domain Organization

**FIGURE 1:**
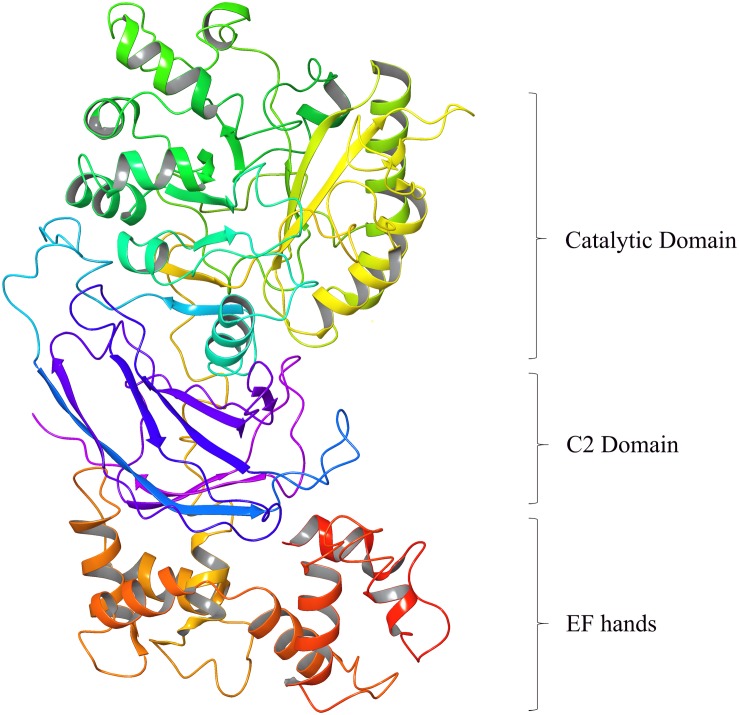
Homology modeling of Human PLCζ (3D ribbon representation) predicted using 1DJI.B PDB entry as template based on a target-template alignment by the Prime homology modeling tool of the Schrödinger Suite (DeLano Scientific LLC, Schrödinger).

Phospholipase C zeta is currently the smallest known mammalian PLC isoform (∼70–75 kDa in size) with the most elementary domain organization (Cox et al., 2002; Saunders et al., 2002; Nomikos et al., 2013a, 2017a). Despite this, PLCζ exhibits uniquely supreme potency in triggering Ca^2+^ oscillations within the fertilizing egg compared to other somatic PLCs. This is attributed to its novel biochemical characteristics, arising from the essential role of its domains that contribute to the unique biological function and mode of regulation of this distinctive PLC isozyme (Nomikos, 2015; Nomikos et al., 2017a). PLCζ domain structure consists of four tandem EF hand domains at the N-terminus, the catalytic X and Y domain in the center of the molecule, followed by a single C2 domain at the C-terminus (Figure 1; Nomikos et al., 2017a). All these domains are common to other PLC isoforms. The X and Y catalytic domains are separated by a short segment, the XY-linker, which through its net positive charge plays an important role in targeting PLCζ to intracellular membranes by direct electrostatic interactions with its negatively charged substrate, PIP_2_ (Nomikos et al., 2007, 2011b). The XY-linker region differs considerably between PLC isozymes (Nomikos et al., 2013a). By contrast, the XY catalytic domain between PLC isoforms is the most highly conserved region (Nomikos, 2015).

The XY domain of PLCζ shares ∼60% sequence similarity to that of all PLCs and is responsible for PIP_2_ hydrolysis (Nomikos et al., 2005). The EF hands are Ca^2+^-binding motifs and in PLCζ these domains play a vital role in its high Ca^2+^ sensitivity compared with the other somatic PLCs, allowing PLCζ to be active at resting Ca^2+^ levels within the egg cytosol, when PLCζ enters after sperm-egg fusion (Nomikos et al., 2005). Additionally, we have demonstrated that the first EF-hand domain of PLCζ, which contains a cluster of basic amino acid residues, plays an essential role together with the XY-linker region, in the interaction of PLCζ with the PIP_2_-containing membranes (Nomikos et al., 2015b). The C-terminal C2 domain of PLCζ, comprising ∼120 amino acid residues is essential for PLCζ function, as targeted deletion or replacement of this domain by the corresponding domain from PLCδ1 abolishes the Ca^2+^-oscillation-inducing activity of PLCζ in eggs, without altering its enzymatic activity or Ca^2+^ sensitivity (Nomikos et al., 2005; Theodoridou et al., 2013). We have provided biochemical evidence that this domain directly interacts with the membrane phospholipids, PI(3)P, and PI(5)P and have suggested that C2 association with these phospholipids may facilitate in the membrane targeting of PLCζ (Theodoridou et al., 2013; Nomikos, 2015).

## PLCζ in Mammalian Sperm

Phospholipase C zeta mRNA has been identified during both early and late stages of spermatogenesis in mice and pigs (Yoneda et al., 2006; Young et al., 2009; Bedford-Guaus et al., 2011; Kaewmala et al., 2012). Specific localization patterns, however, remain elusive in the literature throughout the various spermatogenic cells within the testes (Kashir et al., 2018). Aarabi et al. (2012) indicated that PLCζ is integrated as part of the acrosome during the Golgi phase of human and mouse spermiogenesis (Aarabi et al., 2012), suggesting that observable PLCζ levels are diminished gradually throughout spermatid elongation (Kashir et al., 2018). PLCζ was originally identified in mouse sperm extract fractions that were able to induce Ca^2+^ release. Subsequent immunofluorescence analysis indicated a post-acrosomal localization for PLCζ; a component of the post-acrosomal sheath (Saunders et al., 2002; Fujimoto et al., 2004; Young et al., 2009). Nevertheless, PLCζ has been identified in the sperm of various mammalian species, and usually tends to be found within the sperm head in distinct subcellular regions, postulating differential functional roles for each population (Amdani et al., 2013; Kashir et al., 2014, 2018).

While in mouse and porcine sperm, PLCζ has been observed mainly at acrosomal and post-acrosomal regions (Fujimoto et al., 2004; Young et al., 2009; Nakai et al., 2011; Kaewmala et al., 2012), in equine sperm, PLCζ was recorded at the acrosome, equatorial segment, and head mid-piece, as well as the principle piece of the flagellum (Bedford-Guaus et al., 2011; Kashir et al., 2018). Several PLCζ populations were observed in humans in multiple studies including the acrosomal, equatorial and post-acrosomal regions of the sperm head, with a potential tail localization (Grasa et al., 2008; Yoon et al., 2008; Young et al., 2009; Kashir et al., 2013b, 2018; Escoffier et al., 2015; Yelumalai et al., 2015; Yeste et al., 2016). While there is consensus regarding PLCζ localization in mouse sperm, the veracity of the multiple populations identified in other mammalian sperm (particularly in humans) remains debated (Nomikos et al., 2017a; Kashir et al., 2018). In human sperm, this variation in PLCζ localization is not only limited to observations between different studies but substantial variability in the PLCζ localization pattern was found even within the same study (Kashir et al., 2013b).

Despite numerous efforts to examine PLCζ localization within mammalian sperm, significant concern surrounds the specificity of the majority of antibodies used to date. More specifically, most antibodies used in the literature are unable to demonstrate a consistent motif of recognizing a single band following immunoblotting of human sperm, often detecting multiple protein bands other than, or in addition to, that of the expected size for native PLCζ protein. Compounded by this non-specificity, multiple groups have identified varying populations between mouse and human sperm, even using the same antibodies, suggesting that varying protocols and the use of different antibodies are the main source of inconsistent results between studies (Grasa et al., 2008; Yoon et al., 2008; Heytens et al., 2009; Kashir et al., 2011a, b, 2013b; Aarabi et al., 2012).

**FIGURE 2:**
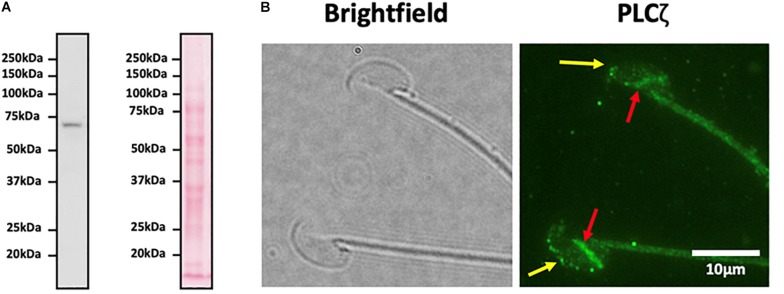
**(A)** Representative immunoblot image obtained using a highly specific anti-PLCζ polyclonal antibody identifying a single protein band corresponding to the molecular weight of native mouse PLCζ (∼74 kDa). Left image indicates immunoblot specificity, while the right image shows ponceau-stained membranes. 1 × 10^6^ mouse sperm were loaded per lane. **(B)** Representative immunofluorescence image illustrating representative localization of native PLCζ in mouse sperm. Image was captured at 100X, and brightfield (left panel) and green fluorescence (right panel; PLCζ) images were obtained. Yellow arrows indicate acrosomal populations, while red arrows indicate post-acrosomal localization of PLCζ in mouse sperm, Scale bar indicates 10 μm.

Addressing such concerns, we recently generated highly epitope-specific PLCζ polyclonal antibodies against human, mouse, and porcine PLCζ, that exhibit high consistency throughout numerous studies for both recombinant and native PLCζ (Nomikos et al., 2013b, 2014, 2015a; Theodoridou et al., 2013). Furthermore, we have also developed specific antigen unmasking/retrieval protocols, which we previously demonstrated are essential to enhance the visualization efficacy of PLCζ in mammalian sperm (Kashir et al., 2017). Using these enhanced protocols and materials, we have identified PLCζ in the acrosomal and post-acrosomal, acrosomal and equatorial, and post-acrosomal and equatorial compartments of mouse, human, and porcine sperm, respectively. Furthermore, we have also consistently observed potential tail localization in all species (Kashir et al., 2017). Figure 2 demonstrates the expression and distribution of PLCζ in mouse sperm using our specific polyclonal antibodies and our recently developed and reported protocols (Kashir et al., 2017). It is now imperative that these specific antibodies and protocols are applied in a systematic manner to examine whether particular localization patterns or profiles of PLCζ exhibit any relationships between male fertility parameters, or indeed between fertility treatment outcomes.

Another intriguing question is how PLCζ, despite its high Ca^2+^-sensitivity and its potent enzymatic activity, is kept in an inactive state within the sperm, especially when it is likely to be present in much higher concentrations in a single spermatozoon than within the fertilizing egg. Indeed, our previous work where it was shown that PLCζ is inactive in somatic cells even at levels over 1000 times that at which it is active in eggs (Phillips et al., 2011), suggests that either PLCζ has an essential binding-partner within the egg, or that other factors within sperm and somatic cells may inhibit its catalytic activity.

## Reduced Expression Levels and Abnormal Forms of Sperm PLCζ Lead to Male Infertility

Infertility is estimated to affect ∼15% of couples, with male infertility affecting ∼7% of men worldwide (Kashir et al., 2010). While genetic causes of male infertility are estimated to underlie ∼30% of such cases (Harton and Tempest, 2012; Jungwirth et al., 2012; Hotaling, 2014), ∼50% of cases of male infertility remain unexplained (Kashir et al., 2018). While most forms of infertility can now be treated via a collection of laboratory techniques collectively termed ART, a number of conditions such as severe male infertility (19–57% of cases) cannot yet been treated (Botezatu et al., 2014). Despite the fact that powerful ART methods such as IVF or ICSI can successfully treat some infertility cases, it is concerning that this is achieved only after several fertility treatment cycles. A significant causative factor may be recurrent implantation failure, which even after fertility treatment leads to infertility (Polanski et al., 2014; Kashir et al., 2018).

Considering the indispensable contribution of PLCζ to fertilization, defects in either egg activation, or in PLCζ protein itself, may underlie conditions of male infertility where fertilization failure occurs. The first evidence came from studies that reported sperm of infertile men, which consistently failed to fertilize eggs following routine IVF or ICSI, and were either unable to induce Ca^2+^ release upon microinjection into mouse eggs, or produced highly abnormal Ca^2+^ transients which were reduced in frequency and amplitude (Yoon et al., 2008; Heytens et al., 2009). Furthermore, such sperm also exhibited reduced or absent levels, as well as abnormal localization patterns, of PLCζ within the sperm head (Heytens et al., 2009; Kashir et al., 2011a, b, 2013b), suggesting that deficiencies in PLCζ protein may underlie currently unknown cases of male factor infertility. In a clinical scenario, in contrast to other causes, complete fertilization failure is attributed to egg activation failure in a species-specific manner (Kashir et al., 2010, 2018).

Moreover, PLCζ gene abrogation in patients diagnosed with egg activation deficiency is now increasingly being reported within the scientific literature. The first two PLCζ mutations were identified in the gene of an infertile male, whose sperm was unable to trigger the normal pattern of Ca^2+^ oscillations, leading to egg activation failure and potentially to his infertility (Heytens et al., 2009; Kashir et al., 2011b, 2012b,2012c). Both mutations were reported within the active catalytic site domains of PLCζ (X and Y), disrupting local protein structural folding to cause reduction of enzymatic activity, subsequently leading to highly abnormal Ca^2+^ transients unable to initiate egg activation (Kashir et al., 2011b). Both mutations were reported to be heterozygous, with one mutation being inherited from the patient’s father and the other from the patient’s mother, indicating for the first time that such maternally inherited loss-of-activity mutations can lead to male infertility (Kashir et al., 2012b, c; Nomikos et al., 2017a). Subsequently, a further mutation in homozygosis was later reported by Escoffier et al. (2016) from two infertile brothers. This mutation is located within the C2 domain of PLCζ (Escoffier et al., 2016). Intriguingly, this PLCζ mutant displayed similar enzymatic activity to wild type PLCζ, but displayed a dramatically reduced relative binding-affinity to PI(3)P and PI(5)P-containing liposomes (Nomikos et al., 2017a). More importantly, this genetic report by Escoffier et al. (2016) and the identification of this novel missense homozygous PLCζ mutation in these infertile brothers after whole exomic sequencing, strongly indicates that absence or defects in PLCζ protein alone is sufficient to prevent human egg activation by the sperm, suggesting that PLCζ is essential for human egg activation and thus human fertilization.

Furthermore, single nucleotide polymorphisms (SNPs) have also been reported by Yoon et al. (2008) and more recently by Ferrer-Vaquer et al. (2016), either within the PLCζ coding sequence or its associated bi-directional promoter in human patients (Nomikos et al., 2017a).

Interestingly, Torra-Massana et al. (2019) very recently reported six new PLCζ mutations after screening an egg activation deficiency group, one of which was previously described (Kashir et al., 2012b, c), in addition to four novel single-nucleotide missense mutations, located in the EF-hands, the X catalytic and C2 domains; while the sixth mutation identified was a frameshift variant, which was predicted to generate a truncated protein at the X-Y linker region (Torra-Massana et al., 2019). While further analysis indicated a potential deleterious effect of some of these mutations upon PLCζ activity within eggs, further biochemical analysis is required to ascertain whether such variants of PLCζ are deleterious as claimed at physiological levels, needing their accurate quantification in sperm and eggs. However, it is now clear that deleterious mutations in PLCζ may be more widespread than previously thought, appearing not only in the catalytic active site, but also in other vital regulatory regions of this essential sperm protein, afflicting its membrane and/or substrate binding, its Ca^2+^ sensitivity, as well as its enzymatic activity.

Despite the relatively high success rate of ICSI in overcoming cases of failed fertilization after IVF, ∼30% of such cases still repeatedly fail ICSI (Flaherty et al., 1998; van der Westerlaken et al., 2005). Low global pregnancy success rates following ART have been attributed to poor embryogenesis following fertility treatment (Fauque et al., 2007; Pelinck et al., 2010). Importantly, such poor embryonic competency can be directly linked to PLCζ and competency of egg activation (Nomikos et al., 2017a; Kashir et al., 2018). Injection of increasing levels of PLCζ in human eggs results in increasing frequencies and amplitudes of Ca^2+^ oscillations (Yamaguchi et al., 2017), which in turn affects subsequent gene expression (Ducibella et al., 2002, 2006). Furthermore, the frequency and amplitude of Ca^2+^ oscillations has been shown to play an important role in compaction, and blastocyst formation (Swann and Ozil, 1994; Miyazaki and Ito, 2006). Finally, taking into consideration that the rate of progression to the 2- and 4-cell stages of human eggs following fertilization has been suggested as an indicator of normal embryogenesis (Wong et al., 2010), PLCζ-driven Ca^2+^ oscillations may not only be required for egg activation, but can also be equally important for subsequent embryogenesis. Thus, abnormalities in sperm PLCζ levels may underlie not only infertility through fertilization failure, but also cases of male subfertility, whereby enough PLCζ may be delivered into the eggs to trigger activation, but prove insufficient for embryonic competence.

## Clinical Applications of PLCζ and Future Directions

Currently, cases of defective egg activation are clinically resolved using assisted egg activation (AOA), involving artificially mediated Ca^2+^ release (Santella and Dale, 2015; Kashir et al., 2018). Ca^2+^ ionophores like ionomycin, calcimycin are currently used to overcome unexplained fertilization failure in couples, who repeatedly fail ICSI cycles (Fawzy et al., 2018; Norozi-Hafshejani et al., 2018). Recently, a meta-analysis indicated that use of Ca^2+^ ionophores significantly improved fertilization and implantation rates in ICSI (Murugesu et al., 2017). However, Ca^2+^ ionophores induce a single Ca^2+^ transient, unlike the endogenous specific physiological pattern of Ca^2+^ oscillations observed during normal fertilization (Rinaudo et al., 1997). In fact, microinjection of human recombinant PLCζ yielded higher blastocyst development rates than Ca^2+^ ionophore treatment (Sanusi et al., 2015). Thus, PLCζ has long represented a physiologically endogenous alternative method to clinically treat cases of egg activation failure/deficiency, which would involve the *in vitro* production of active, purified versions of human recombinant PLCζ protein. Indeed, following initial difficulties, multiple studies made advancements in the production of such a desired product (Kashir et al., 2011b; Yoon et al., 2012), culminating in efforts by Nomikos et al. (2013b) who made a significant breakthrough by generating purified, highly active recombinant PLCζ, capable of inducing physiological patterns of Ca^2+^ oscillations following microinjection into mouse and human eggs (Nomikos et al., 2013b). More importantly, recombinant PLCζ was able to effectively rescue failed egg activation in a prototype of male infertility (Nomikos et al., 2013b). Further attempts to develop the use of recombinant PLCζ protein as a therapeutic agent in a clinical setting are currently in progress, in order to eliminate any potential cytotoxic effects during embryonic development and confirm the overall safety of exogenous PLCζ on the subsequent offspring.

Finally, PLCζ not only represents a promising clinical therapeutic agent, but also a potentially powerful diagnostic biomarker, which may help in determining the criteria and requirements of the fertility treatment of male patients, significantly decreasing the number of cycles needed for a successful pregnancy to occur. Taking into consideration that PLCζ analysis might be beneficial to identify not only cases of ICSI-failure but also cases of male subfertility, a simple immunocytological approach to routinely examine PLCζ protein in sperm is currently widely regarded as a cost-effective approach, which could be easily applied by the majority of IVF clinics worldwide. However, it is essential that studies focus on the reliable investigation of PLCζ parameters in relation to sperm health, using robust protocols and ultra-specific antibodies, before such clinical promise can be achieved. Indeed, significant issues remain regarding such analyses, particularly in humans, where PLCζ antibodies used recognize multiple protein bands in addition to the full-length PLCζ protein. The same antibodies have also been used to identify different immunoblotting profiles (for detailed review see Kashir et al., 2018). Predictably, such shortcomings have led to conflicting results between the association of specific PLCζ localization patterns and quantification levels.

## Ethics Statement

Use of mouse sperm cells was carried out in accordance with the principles of the Basel Declaration and recommendations of the Animal Care and Use Committee (ACUC) at the Office of Research Affairs (ORA) at the King Faisal Specialist Hospital and Research Center, Riyadh, Saudi Arabia. The protocols utilized for the relevant study (RAC-216004) were approved by the ACUC.

## Author Contributions

AS, JK, AT, and MN prepared the first draft of the manuscript, which was revised and approved by all authors.

## Conflict of Interest

The authors declare that the research was conducted in the absence of any commercial or financial relationships that could be construed as a potential conflict of interest.

## Abbreviations

ART: assisted reproductive technology
Ca^2+^: calcium
ICSI: intracytoplasmic sperm injection
InsP_3_: inositol 1,4,5-trisphosphate
PI(3)P: phosphatidylinositol 3-phosphate
PI(5P): phosphatidylinositol 5-phosphate
PIP_2_: phosphatidylinositol 4,5-bisphosphate
PLC ζ: phospholipase C zeta.

## References

[B1] Aarabi M. Qin Z. Xu W. Mewburn J. Oko R. (2010). Sperm-borne protein, PAWP, initiates zygotic development in Xenopus laevis by eliciting intracellular calcium release. *Mol. Reprod. Dev.* 77 249–256. 10.1002/mrd.21140 20017143

[B2] Aarabi M. Yu Y. Xu W. Tse M. Y. Pang S. C. Yi Y. J. (2012). The testicular and epididymal expression profile of PLCzeta in mouse and human does not support its role as a sperm-borne oocyte activating factor. *PLoS One* 7:e33496. 10.1371/journal.pone.0033496 22428063 PMC3299792

[B3] Amdani S. N. Jones C. Coward K. (2013). Phospholipase C zeta (PLCzeta): oocyte activation and clinical links to male factor infertility. *Adv. Biol. Regul.* 53 292–308. 10.1016/j.jbior.2013.07.005 23916605

[B4] Bedford-Guaus S. J. McPartlin L. A. Xie J. Westmiller S. L. Buffone M. G. Roberson M. S. (2011). Molecular cloning and characterization of phospholipase C zeta in equine sperm and testis reveals species-specific differences in expression of catalytically active protein. *Biol. Reprod.* 85 78–88. 10.1095/biolreprod.110.089466 21389344

[B5] Botezatu A. Socolov R. Socolov D. Iancu I. V. Anton G. (2014). Methylation pattern of methylene tetrahydrofolate reductase and small nuclear ribonucleoprotein polypeptide N promoters in oligoasthenospermia: a case-control study. *Reprod. Biomed Online* 28 225–231. 10.1016/j.rbmo.2013.10.010 24365028

[B6] Cheng Y. Zhong Z. Latham K. E. (2012). Strain-specific spontaneous activation during mouse oocyte maturation. *Fertil. Steril.* 98 200–206. 10.1016/j.fertnstert.2012.03.060 22584025 PMC3389194

[B7] Cox L. J. Larman M. G. Saunders C. M. Hashimoto K. Swann K. Lai F. A. (2002). Sperm phospholipase Czeta from humans and cynomolgus monkeys triggers Ca2+ oscillations, activation and development of mouse oocytes. *Reproduction* 124 611–623. 10.1530/rep.0.1240611 12416999

[B8] Cran D. G. Moor R. M. Irvine R. F. (1988). Initiation of the cortical reaction in hamster and sheep oocytes in response to inositol trisphosphate. *J. Cell Sci.* 91(Pt 1), 139–144. 3267017 10.1242/jcs.91.1.139

[B9] Ducibella T. Huneau D. Angelichio E. Xu Z. Schultz R. M. Kopf G. S. (2002). Egg-to-embryo transition is driven by differential responses to Ca(2+) oscillation number. *Dev. Biol.* 250 280–291. 10.1006/dbio.2002.0788 12376103

[B10] Ducibella T. Schultz R. M. Ozil J. P. (2006). Role of calcium signals in early development. *Semin. Cell Dev. Biol.* 17 324–332. 10.1016/j.semcdb.2006.02.010 16580237

[B11] Escoffier J. Lee H. C. Yassine S. Zouari R. Martinez G. Karaouzene T. (2016). Homozygous mutation of PLCZ1 leads to defective human oocyte activation and infertility that is not rescued by the WW-binding protein PAWP. *Hum. Mol. Genet.* 25 878–891. 10.1093/hmg/ddv617 26721930 PMC4754041

[B12] Escoffier J. Yassine S. Lee H. C. Martinez G. Delaroche J. Coutton C. (2015). Subcellular localization of phospholipase Czeta in human sperm and its absence in DPY19L2-deficient sperm are consistent with its role in oocyte activation. *Mol. Hum. Reprod.* 21 157–168. 10.1093/molehr/gau098 25354701 PMC4311148

[B13] Fauque P. Leandri R. Merlet F. Juillard J. C. Epelboin S. Guibert J. (2007). Pregnancy outcome and live birth after IVF and ICSI according to embryo quality. *J. Assist. Reprod. Genet.* 24 159–165. 10.1007/s10815-007-9115-z 17340190 PMC3455053

[B14] Fawzy M. Emad M. Mahran A. Sabry M. Fetih A. N. Abdelghafar H. (2018). Artificial oocyte activation with SrCl2 or calcimycin after ICSI improves clinical and embryological outcomes compared with ICSI alone: results of a randomized clinical trial. *Hum. Reprod.* 33 1636–1644. 10.1093/humrep/dey258 30099496

[B15] Ferrer-Vaquer A. Barragan M. Freour T. Vernaeve V. Vassena R. (2016). PLCzeta sequence, protein levels, and distribution in human sperm do not correlate with semen characteristics and fertilization rates after ICSI. *J. Assist. Reprod. Genet.* 33 747–756. 10.1007/s10815-016-0718-0 27138933 PMC4889489

[B16] Flaherty S. P. Payne D. Matthews C. D. (1998). Fertilization failures and abnormal fertilization after intracytoplasmic sperm injection. *Hum. Reprod.* 13(Suppl. 1), 155–164. 10.1093/humrep/13.suppl_1.155 9663780

[B17] Fujimoto S. Yoshida N. Fukui T. Amanai M. Isobe T. Itagaki C. (2004). Mammalian phospholipase Czeta induces oocyte activation from the sperm perinuclear matrix. *Dev. Biol.* 274 370–383. 10.1016/j.ydbio.2004.07.025 15385165

[B18] Grasa P. Coward K. Young C. Parrington J. (2008). The pattern of localization of the putative oocyte activation factor, phospholipase Czeta, in uncapacitated, capacitated, and ionophore-treated human spermatozoa. *Hum. Reprod.* 23 2513–2522. 10.1093/humrep/den280 18653671

[B19] Hachem A. Godwin J. Ruas M. Lee H. C. Ferrer Buitrago M. Ardestani G. (2017). PLCzeta is the physiological trigger of the Ca(2+) oscillations that induce embryogenesis in mammals but conception can occur in its absence. *Development* 144 2914–2924. 10.1242/dev.150227 28694258 PMC5592814

[B20] Harada Y. Matsumoto T. Hirahara S. Nakashima A. Ueno S. Oda S. (2007). Characterization of a sperm factor for egg activation at fertilization of the newt Cynops pyrrhogaster. *Dev. Biol.* 306 797–808. 10.1016/j.ydbio.2007.04.019 17499700

[B21] Harton G. L. Tempest H. G. (2012). Chromosomal disorders and male infertility. *Asian. J. Androl.* 14 32–39. 10.1038/aja.2011.66 22120929 PMC3735152

[B22] Heytens E. Parrington J. Coward K. Young C. Lambrecht S. Yoon S. Y. (2009). Reduced amounts and abnormal forms of phospholipase C zeta (PLCzeta) in spermatozoa from infertile men. *Hum. Reprod.* 24 2417–2428. 10.1093/humrep/dep207 19584136

[B23] Hotaling J. M. (2014). Genetics of male infertility. *Urol. Clin. North Am.* 41 1–17. 10.1016/j.ucl.2013.08.009 24286764

[B24] Jones K. T. (1998). Ca2+ oscillations in the activation of the egg and development of the embryo in mammals. *Int. J. Dev. Biol.* 42 1–10. 9496781

[B25] Jones K. T. (2018). Mammalian sperm contain two factors for calcium release and egg activation: phospholipase C zeta and a cryptic activating factor. *Mol. Hum. Reprod.* 24 465–468. 10.1093/molehr/gay038 30257016

[B26] Jones K. T. Cruttwell C. Parrington J. Swann K. (1998). A mammalian sperm cytosolic phospholipase C activity generates inositol trisphosphate and causes Ca2+ release in sea urchin egg homogenates. *FEBS Lett.* 437 297–300. 10.1016/s0014-5793(98)01254-x 9824311

[B27] Jungwirth A. Giwercman A. Tournaye H. Diemer T. Kopa Z. Dohle G. (2012). European association of urology guidelines on male infertility: the 2012 update. *Eur. Urol.* 62 324–332. 10.1016/j.eururo.2012.04.048 22591628

[B28] Kaewmala K. Uddin M. J. Cinar M. U. Grosse-Brinkhaus C. Jonas E. Tesfaye D. (2012). Investigation into association and expression of PLCz and COX-2 as candidate genes for boar sperm quality and fertility. *Reprod. Domest. Anim.* 47 213–223. 10.1111/j.1439-0531.2011.01831.x 21752105

[B29] Kashir J. Buntwal L. Nomikos M. Calver B. L. Stamatiadis P. Ashley P. (2017). Antigen unmasking enhances visualization efficacy of the oocyte activation factor, phospholipase C zeta, in mammalian sperm. *Mol. Hum. Reprod.* 23 54–67. 10.1093/molehr/gaw073 27932551

[B30] Kashir J. Deguchi R. Jones C. Coward K. Stricker S. A. (2013a). Comparative biology of sperm factors and fertilization-induced calcium signals across the animal kingdom. *Mol. Reprod. Dev.* 80 787–815. 10.1002/mrd.22222 23900730

[B31] Kashir J. Heindryckx B. Jones C. De Sutter P. Parrington J. Coward K. (2010). Oocyte activation, phospholipase C zeta and human infertility. *Hum. Reprod. Update* 16 690–703. 10.1093/humupd/dmq018 20573804

[B32] Kashir J. Heynen A. Jones C. Durrans C. Craig J. Gadea J. (2011a). Effects of cryopreservation and density-gradient washing on phospholipase C zeta concentrations in human spermatozoa. *Reprod. Biomed Online* 23 263–267. 10.1016/j.rbmo.2011.04.006 21665540

[B33] Kashir J. Jones C. Coward K. (2012a). Calcium oscillations, oocyte activation, and phospholipase C zeta. *Adv. Exp. Med. Biol.* 740 1095–1121. 10.1007/978-94-007-2888-2_50 22453985

[B34] Kashir J. Jones C. Lee H. C. Rietdorf K. Nikiforaki D. Durrans C. (2011b). Loss of activity mutations in phospholipase C zeta (PLCzeta) abolishes calcium oscillatory ability of human recombinant protein in mouse oocytes. *Hum. Reprod.* 26 3372–3387. 10.1093/humrep/der336 22010140 PMC3212881

[B35] Kashir J. Jones C. Mounce G. Ramadan W. M. Lemmon B. Heindryckx B. (2013b). Variance in total levels of phospholipase C zeta (PLC-zeta) in human sperm may limit the applicability of quantitative immunofluorescent analysis as a diagnostic indicator of oocyte activation capability. *Fertil. Steril.* 99 107–117. 10.1016/j.fertnstert.2012.09.001 23040527

[B36] Kashir J. Konstantinidis M. Jones C. Heindryckx B. De Sutter P. Parrington J. (2012b). Characterization of two heterozygous mutations of the oocyte activation factor phospholipase C zeta (PLCzeta) from an infertile man by use of minisequencing of individual sperm and expression in somatic cells. *Fertil. Steril.* 98 423–431. 10.1016/j.fertnstert.2012.05.002 22633260

[B37] Kashir J. Konstantinidis M. Jones C. Lemmon B. Lee H. C. Hamer R. (2012c). A maternally inherited autosomal point mutation in human phospholipase C zeta (PLCzeta) leads to male infertility. *Hum. Reprod.* 27 222–231. 10.1093/humrep/der384 22095789 PMC3241606

[B38] Kashir J. Nomikos M. Lai F. A. (2018). Phospholipase C zeta and calcium oscillations at fertilisation: the evidence, applications, and further questions. *Adv. Biol. Regul.* 67 148–162. 10.1016/j.jbior.2017.10.012 29108881

[B39] Kashir J. Nomikos M. Lai F. A. Swann K. (2014). Sperm-induced Ca2+ release during egg activation in mammals. *Biochem. Biophys. Res. Commun.* 450 1204–1211. 10.1016/j.bbrc.2014.04.078 24769204

[B40] Knott J. G. Kurokawa M. Fissore R. A. Schultz R. M. Williams C. J. (2005). Transgenic RNA interference reveals role for mouse sperm phospholipase Czeta in triggering Ca2+ oscillations during fertilization. *Biol. Reprod.* 72 992–996. 10.1095/biolreprod.104.036244 15601914

[B41] Kouchi Z. Shikano T. Nakamura Y. Shirakawa H. Fukami K. Miyazaki S. (2005). The role of EF-hand domains and C2 domain in regulation of enzymatic activity of phospholipase Czeta. *J. Biol. Chem.* 280 21015–21021. 10.1074/jbc.M412123200 15790568

[B42] Limatola N. Vasilev F. Chun J. T. Santella L. (2019a). Altered actin cytoskeleton in ageing eggs of starfish affects fertilization process. *Exp. Cell Res.* 381 179–190. 10.1016/j.yexcr.2019.05.007 31082375

[B43] Limatola N. Vasilev F. Chun J. T. Santella L. (2019b). Sodium-mediated fast electrical depolarization does not prevent polyspermic fertilization in *Paracentrotus lividus* eggs. *Zygote* 27 241–249. 10.1017/S0967199419000364 31397235

[B44] Malcuit C. Kurokawa M. Fissore R. A. (2006). Calcium oscillations and mammalian egg activation. *J. Cell Physiol.* 206 565–573. 10.1002/jcp.20471 16155907

[B45] Miyazaki S. Ito M. (2006). Calcium signals for egg activation in mammals. *J. Pharmacol. Sci.* 100 545–552. 10.1254/jphs.cpj06003x 16799264

[B46] Miyazaki S. Yuzaki M. Nakada K. Shirakawa H. Nakanishi S. Nakade S. (1992). Block of Ca2+ wave and Ca2+ oscillation by antibody to the inositol 1,4,5-trisphosphate receptor in fertilized hamster eggs. *Science* 257 251–255. 10.1126/science.1321497 1321497

[B47] Murugesu S. Saso S. Jones B. P. Bracewell-Milnes T. Athanasiou T. Mania A. (2017). Does the use of calcium ionophore during artificial oocyte activation demonstrate an effect on pregnancy rate? A meta-analysis. *Fertil. Steril.* 108, 468–462. 10.1016/j.fertnstert.2017.06.029 28865547

[B48] Nakai M. Ito J. Sato K. Noguchi J. Kaneko H. Kashiwazaki N. (2011). Pre-treatment of sperm reduces success of ICSI in the pig. *Reproduction* 142 285–293. 10.1530/rep-11-0073 21610169

[B49] Nomikos M. (2015). Novel signalling mechanism and clinical applications of sperm-specific PLCzeta. *Biochem. Soc,. Trans.* 43 371–376. 10.1042/BST20140291 26009178

[B50] Nomikos M. Blayney L. M. Larman M. G. Campbell K. Rossbach A. Saunders C. M. (2005). Role of phospholipase C-zeta domains in Ca2+-dependent phosphatidylinositol 4,5-bisphosphate hydrolysis and cytoplasmic Ca2+ oscillations. *J. Biol. Chem.* 280 31011–31018. 10.1074/jbc.M500629200 16000311

[B51] Nomikos M. Elgmati K. Theodoridou M. Calver B. L. Cumbes B. Nounesis G. (2011a). Male infertility-linked point mutation disrupts the Ca2+ oscillation-inducing and PIP(2) hydrolysis activity of sperm PLCzeta. *Biochem. J.* 434 211–217. 10.1042/BJ20101772 21204786 PMC3195387

[B52] Nomikos M. Elgmati K. Theodoridou M. Calver B. L. Nounesis G. Swann K. (2011b). Phospholipase Czeta binding to PtdIns(4,5)P2 requires the XY-linker region. *J. Cell Sci.* 124(Pt 15), 2582–2590. 10.1242/jcs.083485 21730019 PMC3138701

[B53] Nomikos M. Kashir J. Lai F. A. (2017a). The role and mechanism of action of sperm PLC-zeta in mammalian fertilisation. *Biochem. J.* 474 3659–3673. 10.1042/BCJ20160521 29061915

[B54] Nomikos M. Kashir J. Swann K. Lai F. A. (2013a). Sperm PLCzeta: from structure to Ca2+ oscillations, egg activation and therapeutic potential. *FEBS Lett.* 587 3609–3616. 10.1016/j.febslet.2013.10.008 24157362

[B55] Nomikos M. Mulgrew-Nesbitt A. Pallavi P. Mihalyne G. Zaitseva I. Swann K. (2007). Binding of phosphoinositide-specific phospholipase C-zeta (PLC-zeta) to phospholipid membranes: potential role of an unstructured cluster of basic residues. *J. Biol. Chem.* 282 16644–16653. 10.1074/jbc.M701072200 17430887

[B56] Nomikos M. Stamatiadis P. Sanders J. R. Beck K. Calver B. L. Buntwal L. (2017b). Male infertility-linked point mutation reveals a vital binding role for the C2 domain of sperm PLCzeta. *Biochem. J.* 474 1003–1016. 10.1042/BCJ20161057 28270562

[B57] Nomikos M. Yu Y. Elgmati K. Theodoridou M. Campbell K. Vassilakopoulou V. (2013b). Phospholipase Czeta rescues failed oocyte activation in a prototype of male factor infertility. *Fertil. Steril.* 99 76–85. 10.1016/j.fertnstert.2012.08.035 22999959 PMC3540263

[B58] Nomikos M. Sanders J. R. Kashir J. Sanusi R. Buntwal L. Love D. (2015a). Functional disparity between human PAWP and PLCzeta in the generation of Ca2+ oscillations for oocyte activation. *Mol. Hum. Reprod.* 21 702–710. 10.1093/molehr/gav034 26116451

[B59] Nomikos M. Sanders J. R. Parthimos D. Buntwal L. Calver B. L. Stamatiadis P. (2015b). Essential role of the EF-hand domain in targeting sperm phospholipase czeta to membrane phosphatidylinositol 4,5-Bisphosphate (PIP2). *J. Biol. Chem.* 290 29519–29530. 10.1074/jbc.M115.658443 26429913 PMC4705952

[B60] Nomikos M. Sanders J. R. Theodoridou M. Kashir J. Matthews E. Nounesis G. (2014). Sperm-specific post-acrosomal WW-domain binding protein (PAWP) does not cause Ca2+ release in mouse oocytes. *Mol. Hum. Reprod.* 20 938–947. 10.1093/molehr/gau056 25057041 PMC4172172

[B61] Nomikos M. Swann K. Lai F. A. (2012). Starting a new life: sperm PLC-zeta mobilizes the Ca2+ signal that induces egg activation and embryo development: an essential phospholipase C with implications for male infertility. *Bioessays* 34 126–134. 10.1002/bies.201100127 22086556

[B62] Norozi-Hafshejani M. Tavalaee M. Azadi L. Bahadorani M. Nasr-Esfahani M. H. (2018). Effects of assisted oocyte activation with calcium- ionophore and strontium chloride on in vitro ICSI outcomes. *Iran. J. Basic Med. Sci.* 21 1109–1117. 10.22038/IJBMS.2018.30422.7331 30483383 PMC6251390

[B63] Nozawa K. Satouh Y. Fujimoto T. Oji A. Ikawa M. (2018). Sperm-borne phospholipase C zeta-1 ensures monospermic fertilization in mice. *Sci. Rep.* 8 1315. 10.1038/s41598-018-19497-6 PMC5778054 29358633

[B64] Pelinck M. J. Hoek A. Simons A. H. Heineman M. J. van Echten-Arends J. Arts E. G. (2010). Embryo quality and impact of specific embryo characteristics on ongoing implantation in unselected embryos derived from modified natural cycle in vitro fertilization. *Fertil. Steril.* 94 527–534. 10.1016/j.fertnstert.2009.03.076 19439287

[B65] Phillips S. V. Yu Y. Rossbach A. Nomikos M. Vassilakopoulou V. Livaniou E. (2011). Divergent effect of mammalian PLCzeta in generating Ca(2)(+) oscillations in somatic cells compared with eggs. *Biochem. J.* 438 545–553. 10.1042/BJ20101581 21692749 PMC3195308

[B66] Polanski L. T. Baumgarten M. N. Quenby S. Brosens J. Campbell B. K. Raine-Fenning N. J. (2014). What exactly do we mean by ‘recurrent implantation failure’? A systematic review and opinion. *Reprod. Biomed Online* 28 409–423. 10.1016/j.rbmo.2013.12.006 24581986

[B67] Puppo A. Chun J. T. Gragnaniello G. Garante E. Santella L. (2008). Alteration of the cortical actin cytoskeleton deregulates Ca2+ signaling, monospermic fertilization, and sperm entry. *PLoS One* 3:e3588. 10.1371/journal.pone.0003588 18974786 PMC2570615

[B68] Rinaudo P. Pepperell J. R. Buradgunta S. Massobrio M. Keefe D. L. (1997). Dissociation between intracellular calcium elevation and development of human oocytes treated with calcium ionophore. *Fertil. Steril.* 68 1086–1092. 10.1016/s0015-0282(97)00406-8 9418702

[B69] Santella L. Dale B. (2015). Assisted yes, but where do we draw the line? *Reprod. Biomed Online* 31 476–478. 10.1016/j.rbmo.2015.06.013 26277587

[B70] Santella L. Limatola N. Chun J. T. (2015). Calcium and actin in the saga of awakening oocytes. *Biochem. Biophys. Res. Commun.* 460 104–113. 10.1016/j.bbrc.2015.03.028 25998739

[B71] Sanusi R. Yu Y. Nomikos M. Lai F. A. Swann K. (2015). Rescue of failed oocyte activation after ICSI in a mouse model of male factor infertility by recombinant phospholipase Czeta. *Mol. Hum. Reprod.* 21 783–791. 10.1093/molehr/gav042 26187950 PMC4586348

[B72] Satouh Y. Ikawa M. (2018). New insights into the molecular events of mammalian fertilization. *Trends Biochem. Sci.* 43 818–828. 10.1016/j.tibs.2018.08.006 30170889 PMC6162164

[B73] Satouh Y. Nozawa K. Ikawa M. (2015). Sperm postacrosomal WW domain-binding protein is not required for mouse egg activation. *Biol. Reprod.* 93 94 10.1095/biolreprod.115.131441 26377222

[B74] Saunders C. M. Larman M. G. Parrington J. Cox L. J. Royse J. Blayney L. M. (2002). PLC zeta: a sperm-specific trigger of Ca(2+) oscillations in eggs and embryo development. *Development* 129 3533–3544. 12117804 10.1242/dev.129.15.3533

[B75] Saunders C. M. Swann K. Lai F. A. (2007). PLCzeta, a sperm-specific PLC and its potential role in fertilization. *Biochem. Soc. Symp.* 23–36. 10.1042/BSS0740023 17233577

[B76] Sette C. Paronetto M. P. Barchi M. Bevilacqua A. Geremia R. Rossi P. (2002). Tr-kit-induced resumption of the cell cycle in mouse eggs requires activation of a Src-like kinase. *EMBO J.* 21 5386–5395. 10.1093/emboj/cdf553 12374739 PMC129085

[B77] Stricker S. A. (1999). Comparative biology of calcium signaling during fertilization and egg activation in animals. *Dev. Biol.* 211 157–176. 10.1006/dbio.1999.9340 10395780

[B78] Swann K. (2018). The role of Ca(2+) in oocyte activation during in vitro fertilization: insights into potential therapies for rescuing failed fertilization. *Biochim. Biophys. Acta Mol. Cell Res.* 1865(11 Pt B), 1830–1837. 10.1016/j.bbamcr.2018.05.003 29746897

[B79] Swann K. Ozil J. P. (1994). Dynamics of the calcium signal that triggers mammalian egg activation. *Int. Rev. Cytol.* 152 183–222. 10.1016/s0074-7696(08)62557-7 8206704

[B80] Swann K. Saunders C. M. Rogers N. T. Lai F. A. (2006). PLCzeta(zeta): a sperm protein that triggers Ca2+ oscillations and egg activation in mammals. *Semin. Cell Dev. Biol.* 17 264–273. 10.1016/j.semcdb.2006.03.009 16730199

[B81] Theodoridou M. Nomikos M. Parthimos D. Gonzalez-Garcia J. R. Elgmati K. Calver B. L. (2013). Chimeras of sperm PLCzeta reveal disparate protein domain functions in the generation of intracellular Ca2+ oscillations in mammalian eggs at fertilization. *Mol. Hum. Reprod.* 19 852–864. 10.1093/molehr/gat070 24152875 PMC3843027

[B82] Torra-Massana M. Cornet-Bartolome D. Barragan M. Durban M. Ferrer-Vaquer A. Zambelli F. (2019). Novel phospholipase C zeta 1 mutations associated with fertilization failures after ICSI. *Hum. Reprod.* 34 1494–1504. 10.1093/humrep/dez094 31347677

[B83] van der Westerlaken L. Helmerhorst F. Dieben S. Naaktgeboren N. (2005). Intracytoplasmic sperm injection as a treatment for unexplained total fertilization failure or low fertilization after conventional in vitro fertilization. *Fertil. Steril.* 83 612–617. 10.1016/j.fertnstert.2004.08.029 15749489

[B84] Wong C. C. Loewke K. E. Bossert N. L. Behr B. De Jonge C. J. Baer T. M. (2010). Non-invasive imaging of human embryos before embryonic genome activation predicts development to the blastocyst stage. *Nat. Biotechnol.* 28 1115–1121. 10.1038/nbt.1686 20890283

[B85] Yamaguchi T. Ito M. Kuroda K. Takeda S. Tanaka A. (2017). The establishment of appropriate methods for egg-activation by human PLCZ1 RNA injection into human oocyte. *Cell Calcium.* 65 22–30. 10.1016/j.ceca.2017.03.002 28320563

[B86] Yelumalai S. Yeste M. Jones C. Amdani S. N. Kashir J. Mounce G. (2015). Total levels, localization patterns, and proportions of sperm exhibiting phospholipase C zeta are significantly correlated with fertilization rates after intracytoplasmic sperm injection. *Fertil. Steril.* 104, 561.e4 567.e4. 10.1016/j.fertnstert.2015.05.018 26054556

[B87] Yeste M. Jones C. Amdani S. N. Yelumalai S. Mounce G. da Silva S. J. (2016). Does advancing male age influence the expression levels and localisation patterns of phospholipase C zeta (PLCzeta) in human sperm? *Sci. Rep.* 6:27543. 10.1038/srep27543 27270687 PMC4897631

[B88] Yoneda A. Kashima M. Yoshida S. Terada K. Nakagawa S. Sakamoto A. (2006). Molecular cloning, testicular postnatal expression, and oocyte-activating potential of porcine phospholipase Czeta. *Reproduction* 132 393–401. 10.1530/rep.1.01018 16940280

[B89] Yoon S. Y. Eum J. H. Lee J. E. Lee H. C. Kim Y. S. Han J. E. (2012). Recombinant human phospholipase C zeta 1 induces intracellular calcium oscillations and oocyte activation in mouse and human oocytes. *Hum. Reprod.* 27 1768–1780. 10.1093/humrep/des092 22456923

[B90] Yoon S. Y. Jellerette T. Salicioni A. M. Lee H. C. Yoo M. S. Coward K. (2008). Human sperm devoid of PLC, zeta 1 fail to induce Ca(2+) release and are unable to initiate the first step of embryo development. *J. Clin. Invest.* 118 3671–3681. 10.1172/JCI36942 18924610 PMC2567839

[B91] Young C. Grasa P. Coward K. Davis L. C. Parrington J. (2009). Phospholipase C zeta undergoes dynamic changes in its pattern of localization in sperm during capacitation and the acrosome reaction. *Fertil. Steril.* 91(5 Suppl.), 2230–2242. 10.1016/j.fertnstert.2008.05.021 18710717

[B92] Yu Y. Saunders C. M. Lai F. A. Swann K. (2008). Preimplantation development of mouse oocytes activated by different levels of human phospholipase C zeta. *Hum. Reprod.* 23 365–373. 10.1093/humrep/dem350 18003622

